# Effects of top electrode material in hafnium-oxide-based memristive systems on highly-doped Si

**DOI:** 10.1038/s41598-020-76333-6

**Published:** 2020-11-11

**Authors:** Sueda Saylan, Haila M. Aldosari, Khaled Humood, Maguy Abi Jaoude, Florent Ravaux, Baker Mohammad

**Affiliations:** 1 System on Chip Center (SoCC), Khalifa University of Science and Technology, P.O. Box 127788, Abu Dhabi, United Arab Emirates; 2 Department of Electrical Engineering and Computer Science, Khalifa University of Science and Technology, P.O. Box 127788, Abu Dhabi, United Arab Emirates; 3 Department of Physics, United Arab Emirates University, P.O. Box 15551, Al Ain, United Arab Emirates; 4 Department of Chemistry, Khalifa University of Science and Technology, P.O. Box 127788, Abu Dhabi, United Arab Emirates

**Keywords:** Nanoscience and technology, Chemical engineering, Electrical and electronic engineering, Electronic materials, Materials for devices, Nanoscale materials, Applied physics, Electronics, photonics and device physics

## Abstract

This work provides useful insights into the development of HfO_2_-based memristive systems with a p-type silicon bottom electrode that are compatible with the complementary metal–oxide–semiconductor technology. The results obtained reveal the importance of the top electrode selection to achieve unique device characteristics. The Ag/HfO_2_/Si devices have exhibited a larger memory window and self-compliance characteristics. On the other hand, the Au/HfO_2_/Si devices have displayed substantial cycle-to-cycle variation in the ON-state conductance. These device characteristics can be used as an indicator for the design of resistive-switching devices in various scenes such as, memory, security, and sensing. The current–voltage (*I*–*V*) characteristics of Ag/HfO_2_/Si and Au/HfO_2_/Si devices under positive and negative bias conditions have provided valuable information on the ON and OFF states of the devices and the underlying resistive switching mechanisms. Repeatable, low-power, and forming-free bipolar resistive switching is obtained with both device structures, with the Au/HfO_2_/Si devices displaying a poorer device-to-device reproducibility. Furthermore, the Au/HfO_2_/Si devices have exhibited N-type negative differential resistance (NDR), suggesting Joule-heating activated migration of oxygen vacancies to be responsible for the SET process in the unstable unipolar mode.

## Introduction

Nonvolatile memories based on resistive random access memories (ReRAMs) have attracted wide attention as an alternative to the conventional Si-based Flash memory devices^1^. However, the use of memristors and memristive systems^2^ is not limited to memories, and their potential for numerous applications such as reconfigurable computing^3^, neuromorphic computing^4,5^, sensing^6–9^, and security^10–12^ has been demonstrated previously. A review of some of the prospective applications of this promising technology can be found in^13^.

The use of p- and n-type doped silicon as bottom electrode (BE) in memristive systems remains an unfulfilled potential to both develop memory, computing, and sensor components using the traditional silicon-based microelectronic technology, and to integrate all these different functionalities monolithically on a single platform. It is also worth mentioning that 3D crossbar arrays employing silicon electrodes have been shown to effectively address the well-known sneak path problem^14–16^ by introducing nonlinearity or asymmetry to the *I*–*V* characteristic of the device through the self-rectifying effect^17^, offering thus an exciting opportunity for the realization of high density, parallel-processing memristor arrays. Moreover, memristive systems based on CMOS compatible functional layers such as silicon oxide^17,18^ and hafnium oxide^8,19,20^ deposited on highly-doped silicon electrodes are promising candidates for establishing future systems such as sensing-immersed-in-computation^21^. Hafnium oxide is highly regarded for being a binary system with only two thermodynamically stable phases in equilibrium: insulating and conducting phases^22^. This stability is anticipated to qualify HfO_2_ for the development of memristive systems exhibiting high reliability and endurance^23^.

The electrodes employed in memristor-based electronic devices may significantly affect the resistive switching (RS) behavior. For metal-oxide thin-film systems that make use of inert metals as electrodes, the switching mode is mostly bipolar^24–28^, although unipolar operation characteristics have also been demonstrated^18^. In some cases, the mode of operation could be changed between bipolar and unipolar by adjusting the operation conditions^29^ or by introducing and tuning the thickness of an oxygen scavenging metal layer^30^.

**Figure 1:**
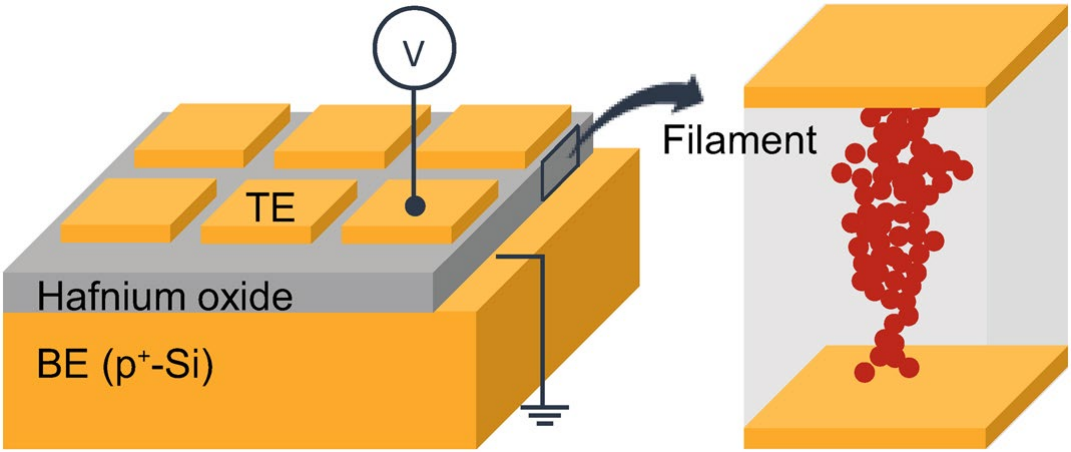
Schematic representation of the fabricated sample and filament formation.

For a memristor architecture with a highly-doped Si bottom electrode, studies concerned with the impact of the top electrode (TE) material on the properties of the memristor are very rare^18,19^. To understand the switching mechanism and to design memristive systems with different functionalities, it is important to know how the SET/RESET operation depends on the choice of top electrode. We have previously reported on the RS behavior of the Ag/HfO_2_/p^+^-Si devices^20^. In this work, the device characteristics have been studied in TE (Ag or Au)/HfO_2_ based memristors on highly-doped p-type Si substrates (illustrated in Fig. 1) by employing electrical characterisation. We have investigated the RS phenomenon in these structures using different electrical biasing schemes, and identified the conditions that induced repeatable high-to-low and low-to-high resistance transitions. Accordingly, we report the important device characteristics such as V_SET_, V_RESET_, R_ON_, R_OFF_, as well as the variation in these characteristics. The TE material has been found to play a key role on the SET/RESET voltage distributions as well as the characteristics of transition from the high resistance state (HRS) to low resistance state (LRS). The results reveal that the Au/HfO_2_/p^+^-Si devices exhibit high cycle-to-cycle and device-to-device variations. It is important and useful to report these unique properties considering the fact that memristive devices find applications in systems where the variability presents a natural opportunity, such as the security applications^10–12,31,32^.

## Experimental results

### Physical characterization

**Figure 2:**
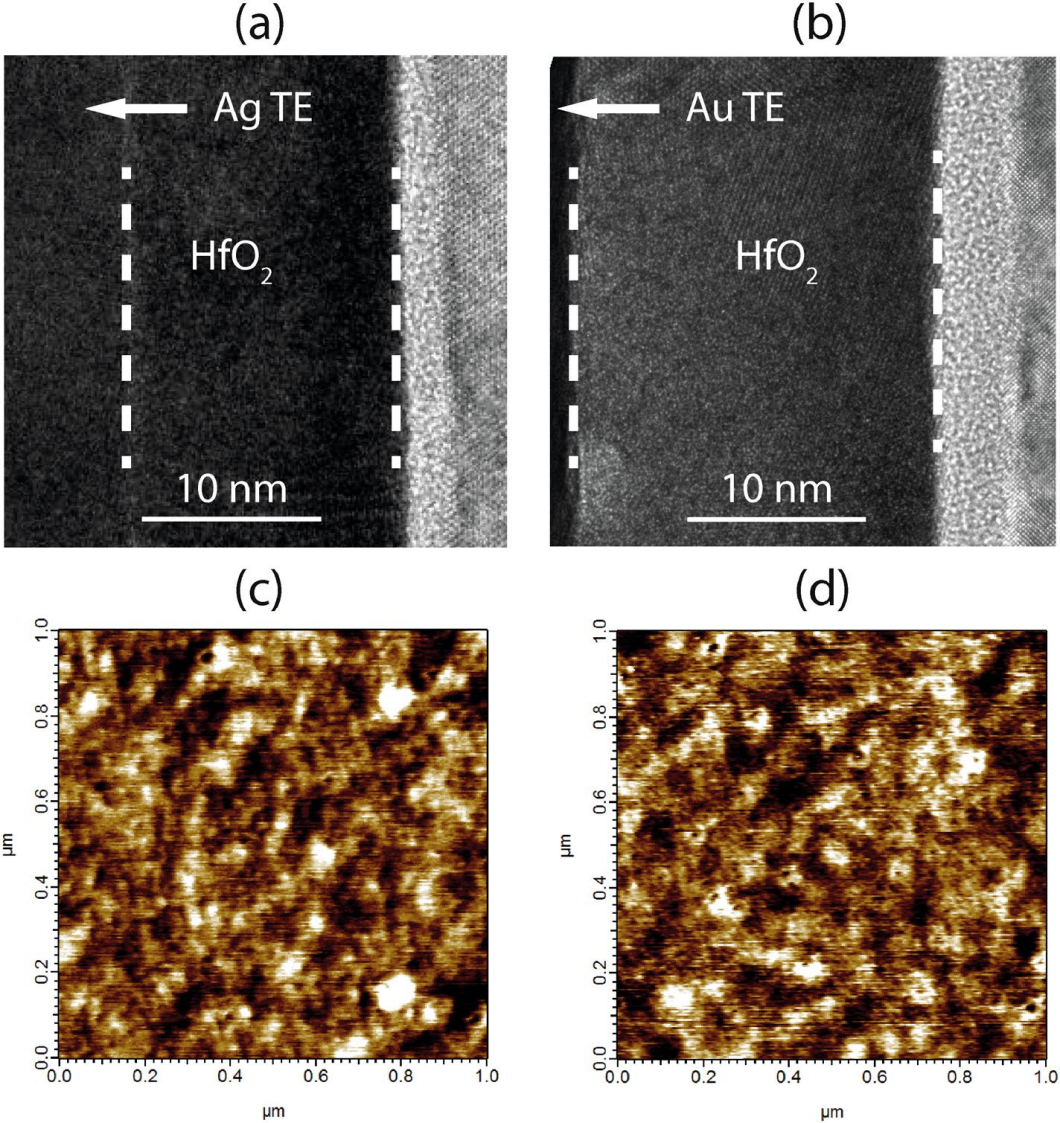
High-resolution TEM images of the fabricated devices with (**a**) Ag TE and (**b**) Au TE. AFM topography scan of the HfO_2_ film used in the (**c**) Ag/HfO_2_/Si stack and (**d**) Au/HfO_2_/Si stack.

HfO_2_ layer thicknesses of around 15 and 20 nm have been obtained for the samples with the Ag and Au TEs, respectively, as seen from the transmission electron microscopy (TEM) images (Fig. 2a,b). The microstructure of the HfO_2_ films used in these samples are very similar as demonstrated in the topographic images of the oxide surface obtained by Atomic Force Microscopy (AFM) (Fig. 2c,d).

### Electrical characterization

The polarity of the electrical bias applied to the memristive stacks may influence the RS characteristics. For instance, the electroforming process for the valence change memory (VCM) based bipolar devices may be polarity dependent due to the built-in asymmetry of the contacts at the top and bottom electrode interfaces as determined by the device design and fabrication procedure^33^. In the case of chemical metallization memory (ECM)^34,35^ based devices, the formation of a filament requires the electrochemical dissolution of an active electrode (usually Ag or Cu), followed by the drift of the injected metal cation clusters toward the counter electrode by a positive voltage applied to the active electrode^26,36,37^. Thus, the comparison of the response of these devices to different biasing schemes may provide valuable information for deciphering the nature of resistive switching in a material system. Observing the current–voltage *(I*–*V)* characteristics of pristine devices through initiating sweeps of opposite polarities on different devices forms the basis of our experimental methodology.

**Figure 3:**
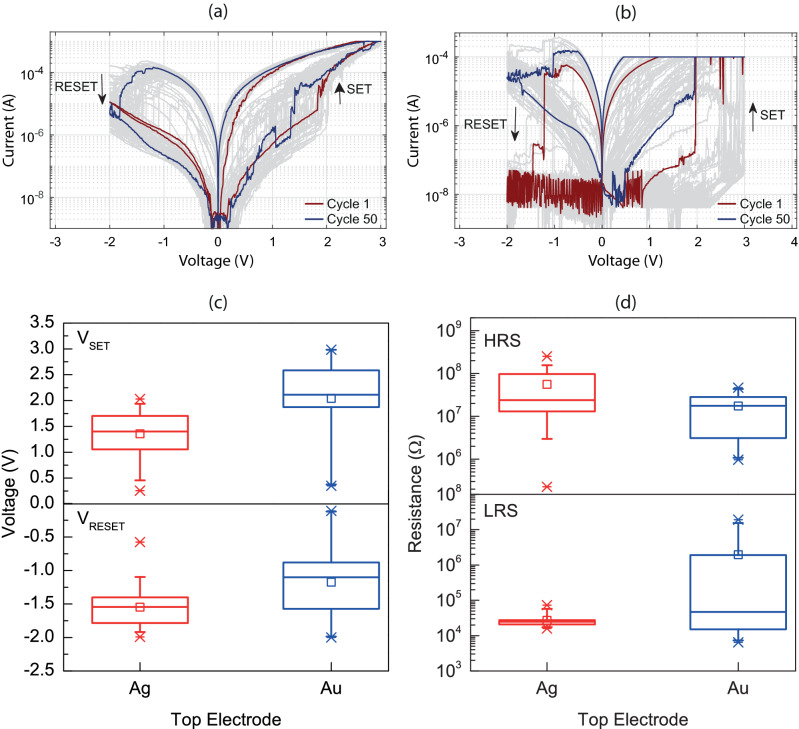
Resistive switching curves with the bias applied on the (**a**) Ag and (**b**) Au top electrodes while the Si bottom electrode is grounded. Arrows indicate the direction of SET and RESET. Statistical distribution of the (**c**) V_SET_-V_RESET_ and (**d**) R_HRS_–R_LRS_ of the TE/HfO_2_/Si devices, where the resistance values were extracted at a voltage of 0.2 V. Boxplot explanation: the box is determined by the 75th and 25th percentiles; the upper and lower whiskers indicate 95th and 5th percentiles, respectively; the horizontal line within the box represents the median; the small box shows the mean value; the upper and lower cross marks indicate the maximum and the minimum, respectively.

The samples employed in this work did not require an initial electroforming step^38^ to produce a repeatable resistive switching behavior. However, as shown in Fig. 3a, the device with the Ag TE exhibited a stable SET/RESET operation only following eight voltage sweep cycles, prior to which it exhibited self-rectifying characteristics^17,20^. Moreover, during the SET operation, the current increased sharply to a level below 10^–4^ A and then increased gradually till the externally applied compliance current (CC), exhibiting the property of self-compliance. The CC was applied to prevent the permanent dielectric breakdown of the metal-oxide and device deterioration^39^. On the other hand, as shown in Fig. 3b, the current flowing through the Au/HfO_2_/Si device reached the CC of 10^–4^ A during the abrupt HRS-to-LRS transition without any self-compliance imposed by the device. Both devices with Ag and Au TEs exhibited a bipolar RS behavior as shown in Fig. 3a,b, respectively, but with different characteristics which are analyzed next. It is worth mentioning that this behavior could not be confirmed with every Au/HfO_2_/Si device tested, presenting a challenge in terms of write/erase reproducibility within the reported electrical testing conditions.

For each of the Ag/HfO_2_/Si and Au/HfO_2_/Si devices, we extracted important device characteristics, such as the V_SET_, V_RESET_, R_LRS_, and R_HRS_ using the data from 50 consecutive SET-RESET sweep cycles presented in Fig. 3a,b. The resistance values were extracted at a voltage of 0.2 V. Figure 3c,d show the statistical distribution of the V_SET_-V_RESET_ and R_HRS_-R_LRS_ for the TE/HfO_2_/Si devices, respectively. It is seen that: (i) the electrical switching in the Au/HfO_2_/Si stack gives a more scattered distribution of the V_RESET_ and R_LRS_ values than in the case of the Ag/HfO_2_/Si stack; (ii) the Au/HfO_2_/Si stack also displays larger V_SET_ and lower V_RESET_ average values. Furthermore, the HRS and LRS resistance values extracted at 0.2 V were used to estimate the off-to-on resistance ratio at each cycle. The Ag/HfO_2_/Si and Au/HfO_2_/Si devices have exhibited a mean R_OFF_/R_ON_ ratio of ~ 2 × 10^3^ and ~ 900, respectively (see Supplementary Fig. S1 online). These results reveal the key role which the TE plays in determining the performance of TE/HfO_2_/Si memristive systems.

**Figure 4:**
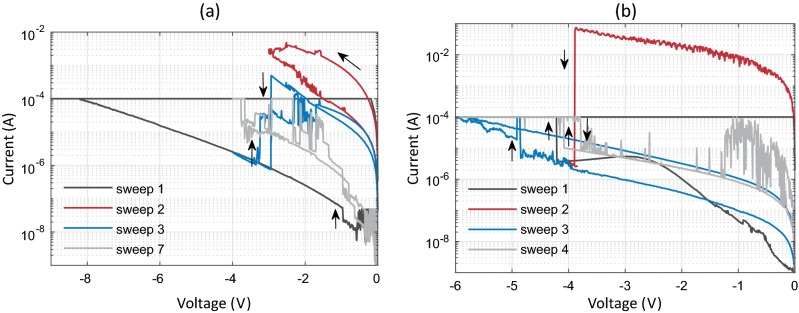
*I*–*V* characteristics recorded during sweeps of negative polarity with the bias applied on the (**a**) Ag and (**b**) Au top electrodes while the Si bottom electrode is grounded. Arrows indicate the direction of SET and RESET.

To further understand the nature of RS behavior of the TE/HfO_2_/Si devices with different TEs, we investigated the *I*–*V* characteristics of pristine devices by applying sweeps of negative polarity, as given in Fig. 4a,b for the Ag and Au top electrodes, respectively. This approach was chosen to eliminate the injection of Ag cations into the hafnium oxide layer though the following electrochemical dissolution half-reaction Ag → Ag^*z*+^ + *ze*^−^ that may take place within the voltage sweep window applied under the positive bias, where the cation valence z is either 1 or 2^40^. It is seen from Fig. 4a,b that: (i) regardless of the type of TE used, the SET and RESET operations occur in the same voltage polarity; (ii) both devices exhibit instability of the SET/RESET operation and the LRS becomes volatile following a couple of sweeps after the first SET (sweep 1), as shown by sweep 7 and sweep 4, respectively, for the Ag/HfO_2_/Si and Au/HfO_2_/Si devices; (iii) for the same stack, the SET and RESET operations occur at around a similar voltage within a range from − 3 to − 4 V and − 4 to − 5 V, respectively, in Ag/HfO_2_/Si and Au/HfO_2_/Si stacks. Another interesting observation worth to be discussed is that the *I*–*V* slope of the first SET (sweep 1) of the Au/HfO_2_/Si stack, shown in Fig. 4b, is negative between ~ − 2.6 and − 4.2 V (V_SET_) corresponding to a voltage-controlled, or N-type, negative differential resistance (NDR)^41^ region followed by an abrupt transition to the LRS.

## Discussion

It is suggested in the literature that, in principle, a voltage controlled NDR is possible through Joule heating and may occur when the resistance increases superlinearly with temperature^42^. Furthermore, it may render the active material electrically unstable. As a result, for reasons of electrical stability, as well as in consistency with the minimum entropy production principle, high field domains separating regions of low field form in initially isotropic materials^41^. Under conditions of negative conductivity which prevail in the case of NDR, normal random fluctuations in the carrier distribution can form large space charge regions leading to domains of high electric field. A permanent nucleation site such as a crystal defect, a doping inhomogeneity, or the cathode itself are considered as sources of the random noise fluctuation^43^. In this regard, the generation of oxygen vacancies in metal-oxide films at the metal-oxide/anode interface by the electrochemical redox reactions^44–46^ may lead to space charge fluctuations. In fact, the generation of oxygen vacancies may be facilitated in the case of N-type NDR in an attempt to reach stability. *Bersuker* et al.^24^ reported a straightforward positive correlation between the temperature increase and oxygen vacancy generation, as well as the importance of a nonuniform temperature profile in promoting the formation of a conductive filament. Similarly, a large internal temperature gradient caused by the Joule heating in the device has been suggested to facilitate the inward migration of oxygen vacancies by the thermophoresis, or Soret effect, to form a conductive filament between the electrodes^47^. It has also been reported that the coexistence of NDR and RS may strongly depend on the ambient relative humidity^48^. The authors suggested that the water molecules could be combined with the oxygen vacancies, thereby accelerating the rupture of oxygen vacancy filaments. The same mechanism could be playing a role in reducing the current as the magnitude of the voltage is increased beyond ~ 2.7 V up to the V_SET_ (Fig. 4b, sweep 1). Therefore, the observation of a voltage-controlled NDR followed by an abrupt transition to the LRS looks very much like the indication of a filament forming process involving oxygen vacancies, although further studies are needed to verify this hypothesis. The proximity in values of the SET and RESET voltages, as seen in Fig. 4b, suggests that the transition from LRS to HRS requires reaching a critical local temperature in the filament.

The mean SET voltage for the bipolar RS of the Ag/HfO_2_/Si device is extracted to be $\stackrel{-}{{V}_{SET}}=1.36 V.$ On the other hand, the SET operation of unipolar RS of the same device required a negative bias of 3 to 4 V in magnitude. The difference in the magnitudes of V_SET_ observed between the bipolar and unipolar modes is significant, suggesting major differences in the mechanism that is responsible for the SET process under opposite bias polarities. From the similarity between the magnitudes of V_SET_ for the Ag/HfO_2_/Si and Au/HfO_2_/Si devices in the unipolar mode, we infer the switching kinetics of the Ag/HfO_2_/Si devices to be also controlled by Joule heating as discussed for the Au/HfO_2_/Si devices above. On the other hand, a low SET voltage exhibited in the bipolar mode suggests a dominant ECM behavior^25^.

Under positive bias to the Ag electrode, Ag preferentially oxidizes to Ag^+^^40^ with the standard redox potential in aqueous solutions given as Ag^+^/Ag (E° = 0.80 V)^49^. This is followed by the migration of Ag^+^ cations through the oxide layer and the nucleation and growth of Ag by reduction at the counter electrode^37^. The standard redox potentials for Au are given as Au^3+^/Au (E° = 1.52 V), and Au^+^/Au (E° = 1.83 V)^49^. The Au-related redox reactions are governed by more positive standard reduction potentials compared to the Ag^+^/Ag system. This indicates that Ag would be more easily oxidized than Au in positive bias^40^. It is worth noting that oxygen is insoluble in gold and will not form a three-dimensional oxide^50^. Therefore, the ionization of Au at the anode interface is not possible through the oxidation of Au atoms at the Au/oxide interface due to the reduction of the oxide, while Ag ions can also be injected from the oxidized Ag.

The mean SET voltage for the bipolar RS of Au/HfO_2_/Si devices is extracted to be $\stackrel{-}{{V}_{SET}}=2.04 V$, which is close to the $\stackrel{-}{{V}_{SET}}$ (1.36 V) of Ag/HfO_2_/Si devices but significantly less than the range of SET voltages (from 4 to 5 V in magnitude) required for their unipolar operation. Considering the small difference between the $\stackrel{-}{{V}_{SET}}$ of the Ag/HfO_2_/Si and Au/HfO_2_/Si devices, it is of interest to investigate the possibility of the metal ion injection and migration to play a role in the switching kinetics of the bipolar RS of the Au/HfO_2_/Si devices. The findings on the diffusion of Au ions in dielectrics reveal differences among various material systems. For example, a study that used Rutherford backscattering (RBS) analysis and capacitance–voltage measurements could not detect the diffusion of Au in SiO_2_ for temperatures up to 600 °C and fields as high as 10^6^ V/cm^51^. Contrarily, the migration of Au cations from the electrode into the oxide was reported in Y/Y_2_O_3_/Au stacks^52^. It should be emphasized that in the aforementioned work, the value of voltage applied to the samples exceeded 10.6 V, which is significantly higher than the value of the maximum positive voltage applied to the Au electrode (i.e., 3 V) in our study. Diffusion of Au atoms in HfO_2_, Al_2_O_3_, and SiO_2_ thin-films has been reported in another study, but only above 500 °C^53^. Furthermore, it is suggested in the literature that the migration rate of metal ions in the oxide films may be strongly influenced by the structural properties of the films such as porosity and density^40^. On the basis of these findings, it is hard to conclude whether Au cations played a role in the bipolar resistive switching exhibited by our devices but it is also hard to rule out the possibility completely.

That said, there is evidence that a large R_OFF_/R_ON_ ratio is more likely to be obtained with the ECM-type devices^54,55^. In this regard, the smaller ratio demonstrated by the Au/HfO_2_/Si devices compared to the Ag/HfO_2_/Si devices suggests that migration of anions, typically oxygen vacancies, may be playing a role in controlling the bipolar switching in the Au/HfO_2_/Si devices.

The statistical distributions of important device characteristics provide critical insights for the design of memristive systems for different applications. In terms of the voltage demand in writing operations, both Ag/HfO_2_/Si (~ 1.36 V) and Au/HfO_2_/Si (~ 2.04 V) devices exhibit a superior performance compared to the silicon-based flash memory technology (16–20 V)^2^. A high on/off conductance ratio is desirable for the memory applications^2^. Therefore, the larger memory window (~ 2 × 10^3^) of the Ag/HfO_2_/Si devices make them suitable candidates for memory applications. Similar results have been previously reported for TiN/HfO_x_/Ti/TiN (> 10^3^)^56^ and Cu/HfO_2_/Pt (10^3^–10^4^)^57^ devices. On the other hand, the R_LRS_ of the Au/HfO_2_/Si devices is observed to be much more dispersed than that of the Ag/HfO_2_/Si. This high cycle-to-cycle variation of the R_LRS_ in the Au/HfO_2_/Si devices (see Supplementary Figs. S2–S4 online) may be exploited for some applications in which the variability presents a natural opportunity. Security applications, a key research area for continued increased performance of future integrated circuits, is one of the areas where variations in device characteristics could be utilized^10,11,31,58^. Furthermore, the self-compliance characteristic, as demonstrated by the Ag/HfO_2_/Si devices, is reported to provide a compelling advantage for practical high-density ReRAMs by reducing the complexity of peripheral circuit design^59^. Also, these devices come with a high endurance potential due to the lack of necessity for electroforming which can cause significant damage to a device^38^.

## Conclusions

In summary, we have investigated the detailed resistive switching behavior of HfO_2_-based devices, fabricated with highly-doped p-type Si BEs and two different TEs of Ag or Au, by means of *I*–*V* measurements. We have demonstrated that the behavior is significantly influenced by the choice of the TE. For instance, the mean ON/OFF ratio decreases from ~ 2 × 10^3^ to ~ 900 as the TE changes from Ag to Au. It was found that both Ag/HfO_2_/Si and Au/HfO_2_/Si devices can exhibit bipolar and unipolar resistive switching modes. Although the bipolar mode was reproducible for the Ag/HfO_2_/Si devices, this behavior could not be confirmed with every Au/HfO_2_/Si device tested within the reported electrical testing conditions. The unipolar mode, which was obtained under the negative bias polarity, was unstable for both device types. During the SET operation of this mode, the Au/HfO_2_/Si devices exhibited N-type NDR which is a phenomenon regarded as difficult to be observed in practice. The associated *I-V* suggests the Joule-heating activated migration of oxygen vacancies and filament formation to be responsible for the SET process in the unipolar mode. On the other hand, a SET voltage much smaller than that of the unipolar mode suggests an ECM-based SET process for the bipolar mode. Further investigations in the future would be necessary to assess the possibility that Au ions diffusing into the HfO_2_ film may be involved in the filament forming process.

## Methods

### Device fabrication

The Ag/HfO_2_/Si and Au/HfO_2_/Si devices investigated in this work were fabricated on two separate Si wafers with a resistivity of 0.004–0.007 Ω-cm (Wafer Works Corp.). For both types of devices, the highly-doped Si substrate served as the BE. The top electrodes (~ 60-nm-thick) were deposited by sputtering through a shadow mask with 2 mm × 2 mm openings. The hafnium oxide thin film in the Ag/HfO_2_/Si stack was deposited by radio frequency (RF) sputtering using an HfO_2_ target of 99.9% purity (AJA International), in Ar atmosphere at 10 mTorr pressure. Similarly, that of the Au/HfO_2_/Si stack was deposited at 4 mTorr pressure in Ar atmosphere by using an HfO_2_ target of 99.99% (Testbourne Ltd.). During the HfO_2_ depositions, the power was set to 100 W and 120 W, respectively, for the Ag/HfO_2_/Si and Au/HfO_2_/Si stacks.

### Physical characterization

TEM imaging of the samples was performed with a FEI Titan S/TEM. The images were obtained in TEM mode at 300 kV. Topographic images of the hafnium oxide films were obtained by a MFP-3D Origin AFM (Asylum Research) using the AC mode imaging in air.

### Electrical characterization

The current–voltage (*I*–*V*) measurements were performed using two parameter analyser systems (Keithley 4200-SCS and Keithley 2450). The Si electrode was electrically grounded in all of the measurements.

## Supplementary information

Supplementary Information.
