# Supplementary material for: Effects of top electrode material in hafnium-oxide-based memristive systems on highly-doped Si
Source: Sci Rep. 2020 Nov 11;10:19541. doi: 10.1038/s41598-020-76333-6 (PMC7658356; doi:10.1038/s41598-020-76333-6)
Supplement: Supplementary file 1 — Supplementary Information. [file 41598_2020_76333_MOESM1_ESM.pdf]

Supplementary Information

Effects of Top Electrode Material in Hafnium-Oxide-Based Memristive Systems on Highly-Doped Si

Sueda Saylan[1,2], Haila M. Aldosari[3], Khaled Humood[1,2], Maguy Abi Jaoude[4,*], Florent Ravaux[2], and Baker Mohammad[1,2,*]

[1]System on Chip Center (SoCC), Khalifa University of Science and Technology, P.O. Box 127788, Abu Dhabi, United Arab Emirates

[2]Department of Electrical Engineering and Computer Science, Khalifa University of Science and Technology, P.O. Box 127788, Abu Dhabi, United Arab Emirates

[3]Department of Physics, United Arab Emirates University, P.O. Box 15551, Al Ain, United Arab Emirates

[4]Department of Chemistry, Khalifa University of Science and Technology, P.O. Box 127788, Abu Dhabi, United Arab Emirates

*Corresponding author. E-mail: baker.mohammad@ku.ac.ae and maguy.abijaoude@ku.ac.ae

S.1 Statistical distribution of the R_{OFF}/R_{ON} ratio in the $Ag/HfO_2/p^+$-Si and $Au/HfO_2/p^+$-Si devices

Figure S1 shows the statistical distribution of R_{OFF}/R_{ON} ratio estimated using the individual HRS and LRS resistance values collected during 50 consecutive write/erase cycles and extracted at 0.2 V.

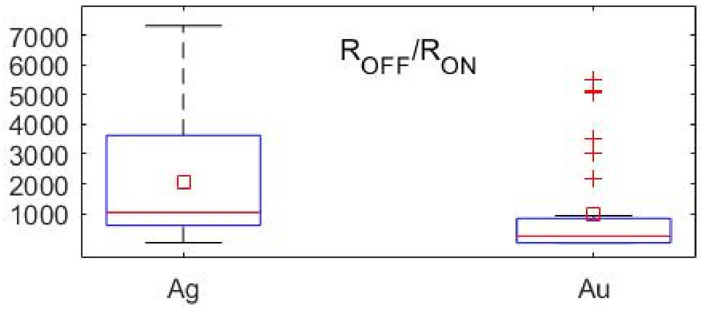

Figure S1. The statistical distribution of R_{OFF}/R_{ON} ratios in the $Ag/HfO_2/p^+$-Si and $Au/HfO_2/p^+$-Si devices.

S.2 Variation in the LRS resistance of Au/HfO_2/p^+-Si devices

Here, we show the statistical distribution of the resistance for two different Au/HfO_2/p^+-Si devices (D1 and D2) obtained using the data from 50 write/erase cycles performed on different days (denoted as Exp1 and Exp2) at room temperature, as well as the statistical distributions presented in Fig. 3(d) as reference. The resistance values have been extracted at a voltage of 0.2 V. As revealed by the data, for both devices (D1 and D2), the variation in the ON-state resistance of the Au/HfO_2/Si devices is reproducible.

For the device D2, we have also performed 50 write/erase cycles at 40 °C (denoted by Exp3) and have extracted the ON-state resistance at 0.2 V. As revealed by the data, the ON-state resistance has exhibited considerable variation at 40 °C as well.

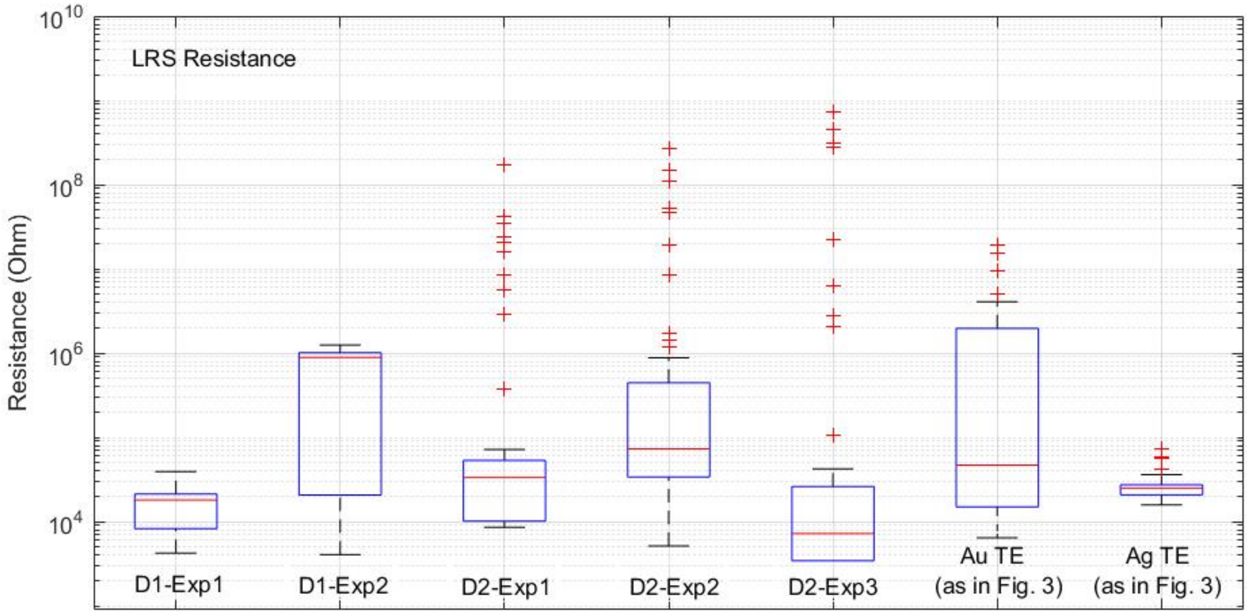

Figure S2. The statistical distribution of LRS resistance (R_{LRS}) extracted based on 50 write/erase cycles performed on different days (denoted as Exp1 and Exp2) at room temperature, using two different Au/HfO_2/Si devices (D1 and D2). The results denoted by Epx3 are for the device D2 collected at 40 °C. The results presented in Fig. 3(d) are also shown for reference.

Time series statistical analyses (TSSA) have shown to be promising in evaluating the variability in the resistive switching characteristics of memristive systems. For instance, these methods have been employed to represent the behavior of the HRS resistance, which linearly increases with the cycle number[1]. Similarly, TSSA have been used to model the set and reset voltages that fulfill the stationarity condition[2]. As seen in Fig. S3(a), the LRS resistance of Au/HfO_2/p^+-Si devices in this work does not follow a linear trend as a function of cycle number. Moreover, a drift in the mean and variance of the LRS resistance data shows up (Fig. S3(b)-(c)), indicating a nonstationary data series.

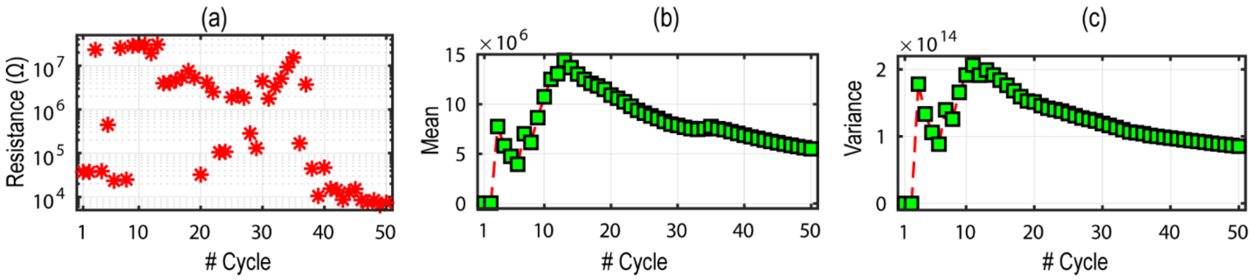

Figure S3. (a) Experimental values of R_{LRS} (at V=0.2 V) versus cycle number, extracted based on 50 write/erase cycles performed using an Au/HfO_2/Si device, along with the (b) mean and (c) variance of the data series.

The influence of externally applied current compliance on the variation of LRS resistance in the Au/HfO_2/Si devices is given below. Figure S4(a)-(b) show the SET *I-V* data for 50 consecutive SET-RESET sweep cycles measured with the same device by applying two different current compliance of 10^{-3} A and 10^{-4} A, respectively. For both cases, the normalized standard deviation[3] (σ_R/μ_R) of LRS resistance has been extracted and presented in Table S1. These normalized standard deviations are also compared with that of the Ag/HfO_2/Si device, which is extracted from the data presented in Fig. 3(a). As seen in Table S1, although the relative spread of R_{LRS} is slightly higher for the case of CC=10^{-4} A; for both cases, the spread in the Au/HfO_2/Si device is substantially larger compared to the spread observed in the Ag/HfO_2/Si device.

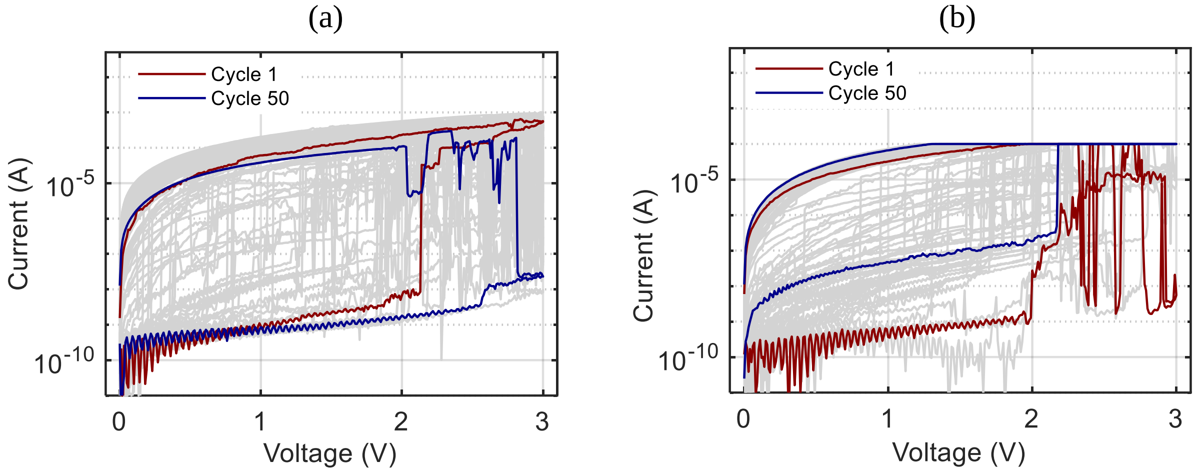

Figure S4. *I-V* curves for an Au/HfO_2/p^+-Si device measured by applying (a) CC=10^{-3} A and (b) CC=10^{-4} A.

Table S1. Relative spread of the LRS resistance σ_R/μ_R.

	Au/HfO_2/p^+-Si (CC=10^{-3} A)	Au/HfO_2/p^+-Si (CC=10^{-4} A)	Ag/HfO_2/p^+-Si (extracted from the data presented in Fig. 3(a))
σ_R/μ_R	5.3	6.9	0.4

References

1 Miranda, E., Mehonic, A., Ng, W. & Kenyon, A. Simulation of Cycle-to-Cycle Instabilities in SiO $ _ {{x}} $-Based ReRAM Devices Using a Self-Correlated Process With Long-Term Variation. *IEEE Electron Device Letters* **40**, 28-31 (2018).

2 Roldan, J., Alonso, F., Aguilera, A., Maldonado, D. & Lanza, M. Time series statistical analysis: A powerful tool to evaluate the variability of resistive switching memories. *J. Appl. Phys.* **125**, 174504 (2019).

3 Ambrogio, S. *et al.* Statistical fluctuations in HfO_x resistive-switching memory: part I-set/reset variability. *IEEE Trans. Electron Devices* **61**, 2912-2919 (2014).
